# Fabrication of Electrospun PAN–CNT–CNC Composite Nanofiber Films for Hydrovoltaic Energy Harvesting

**DOI:** 10.3390/polym18192430

**Published:** 2026-10-06

**Authors:** Hee Chang Jeon, Woohyuk Jang, Jiseon Yun, Sein Min, Joong Yeon Lim, Young-Seong Kim

**Affiliations:** Department of Mechanical, Robotics and Energy Engineering, Dongguk University, Jung-gu, Seoul 04620, Republic of Korea; hcjeon@dongguk.edu (H.C.J.); jangwh21270@dgu.ac.kr (W.J.); ilyoung0430@dgu.ac.kr (J.Y.); molang0721@dgu.ac.kr (S.M.)

**Keywords:** electrospinning, polyacrylonitrile, carbon nanotube, cellulose nanocrystal, ionovoltaic effect, hydrovoltaic energy harvesting, electric double layer

## Abstract

Water-induced electrical responses were investigated using porous electrospun PAN-based composite membranes containing CNTs and CNCs. Following the injection of 20 μL of a 2 wt% NaCl solution, transient open-circuit voltage responses were observed from the PAN, PAN–CNT, and PAN–CNT–CNC devices, with maximum values of approximately 47, 42, and 68 mV, respectively, in the tested devices. Three successive injection–drying cycles demonstrated repeated transient responses within individual devices but did not establish device-to-device reproducibility or long-term stability. Raman and FTIR analyses supported the incorporation of CNTs and the modification of the water-interacting environment following CNC addition, respectively. The voltage waveform depended on the liquid type, NaCl concentration, and wetting–drying history, including a transient reverse-voltage response during drying. These results provide a proof-of-concept observation of wetting–drying-dependent electrical behavior in electrospun PAN-based porous membranes. Load-dependent power, long-term durability, and device-to-device reproducibility remain to be established.

## 1. Introduction

The growing demand for sustainable and distributed energy sources has accelerated the development of energy-harvesting technologies that convert ambient resources into usable electrical energy. Although fossil fuels have supported industrial development and economic growth since the Industrial Revolution, their finite availability, greenhouse-gas emissions, and associated environmental impacts have increased the need for cleaner and more sustainable energy technologies [1]. Renewable-energy systems based on solar radiation, wind, ocean waves, and geothermal heat have consequently been developed as alternatives to conventional fossil-fuel-based power generation [2,3,4]. In parallel, energy harvesting from commonly available environmental sources, including solar light, thermal gradients, vibration, mechanical pressure, humidity, and fluid motion, has attracted considerable attention as a potential power source for distributed sensors, wearable electronics, and low-power electronic devices [5,6,7].

Among the various ambient energy sources, water is particularly attractive because it is abundant and can participate in energy-conversion processes through evaporation, infiltration, flow, moisture adsorption, and ion transport at solid interfaces [8]. Electrolyte-containing water sources, such as human sweat and seawater, are especially relevant because dissolved ions can contribute to interfacial charge redistribution and directional ion transport. Human sweat may therefore serve as a readily accessible energy source for wearable electronic devices [9,10,11], whereas seawater, which covers approximately 70% of the Earth’s surface, represents an abundant and sustainable electrolyte resource [12,13,14]. However, conventional marine energy technologies primarily utilize macroscopic potential or mechanical energy, including tidal height differences, ocean currents, and wave motion [15]. These systems generally require large-scale infrastructure and are strongly constrained by geographical location, installation area, construction cost, and facility scale [16,17]. Consequently, compact energy-harvesting systems capable of directly converting water–solid interfacial interactions into electrical energy without large mechanical facilities are highly desirable.

Water infiltration and capillary transport have been extensively investigated in physical chemistry, hydrology, and geology [18]. More recently, these transport phenomena have been utilized to generate electricity through interactions between water and charged solid surfaces. In particular, when capillary transport occurs within nano- or microscale pores, the movement of water and dissolved ions along the solid interface can generate an electrical potential [19]. Several mechanisms have been proposed to explain water-induced electricity generation. A representative mechanism is the streaming potential produced by the directional transport of mobile ions through charged capillary channels. Proton transport associated with capillary water flow has also been suggested to generate a pseudo-streaming current. However, devices based on these mechanisms commonly require the simultaneous maintenance of continuous capillary flow and evaporation, which complicates device design and long-term operation [20]. Other reported mechanisms involve selective ion adsorption on solid surfaces, ion-concentration-dependent output, and changes in voltage polarity according to the direction of water infiltration. These observations indicate that water-induced electricity generation can arise from complex dynamic interactions among water molecules, dissolved ions, surface charges, and charge carriers at the water–solid interface [18].

Despite these advances, many previously reported water-enabled electricity-generating materials remain limited by insufficient mechanical flexibility, restricted active area, complex fabrication, and difficulties in scalable device integration [21]. Electrospinning offers a potentially scalable route for fabricating porous, flexible, lightweight, and large-area nanofiber films [22]. Polyacrylonitrile (PAN) is particularly suitable as a supporting polymer because of its chemical stability, mechanical flexibility, processability, and compatibility with electrospinning [23]. The interconnected porous structure of electrospun PAN nanofibers provides a high surface-to-volume ratio and pathways for liquid absorption through microscale and nanoscale voids. The resulting interfacial area may facilitate contact among the membrane, water molecules, and dissolved ions. Similar surface-adsorption-related changes in electrical behavior have been reported in other high-surface-area nanostructured materials [24,25,26]. These characteristics motivated the investigation of electrospun PAN nanofiber films as a platform for NaCl-solution-induced transient electrical responses.

To tailor the functional properties of the PAN nanofiber film, carbon nanotubes (CNTs) and cellulose nanocrystals (CNCs) were introduced into the polymer matrix. CNTs possess high electrical conductivity and favorable charge-transfer characteristics and have been widely investigated as potentially conductive fillers in polymer composites [27]. CNTs have also been employed as solid-state electron mediators in other interfacial energy-conversion systems [28]. Although the operating mechanisms of those systems differ from the saline-infiltration-induced electrical response investigated here, these previously reported properties provided the material-design rationale for incorporating CNTs into the PAN-based formulation. However, they do not demonstrate that 0.1 wt% CNTs formed a continuous conductive network or improved charge transport in the present nanofiber film.

CNCs are cellulose-derived nanomaterials with low density, biodegradability, favorable mechanical properties, and compatibility with polymer matrices [29]. Their hydroxyl-rich surfaces can interact with aqueous environments, and nanocellulose-based materials have been investigated in biomedical systems, coatings, food packaging, flexible electronics, energy-harvesting devices, and sensors [30]. These characteristics motivated the incorporation of CNCs to examine whether their addition to the PAN–CNT formulation altered the water-interacting behavior and transient electrical response of the nanofiber film. However, because quantitative wettability and liquid-transport measurements were not performed, enhanced hydrophilicity or uniform NaCl-solution infiltration cannot be established from the present results.

Previous studies on cellulose–carbon nanomaterial composites have reported composition- and structure-dependent changes in electrical transport, surface interactions, and interfacial behavior [31,32]. Cellulose-containing hybrid interfaces have also been investigated in other energy-conversion systems [33]. These studies provide a general material-design basis for examining a CNT- and CNC-containing PAN formulation but do not constitute direct evidence of the functions of the additives in the present device. In particular, because a PAN–CNC control was not included, the independent contribution of CNCs and any synergistic interaction between CNTs and CNCs cannot be determined.

Accordingly, three types of porous electrospun nanofiber films—pristine PAN, PAN–CNT, and PAN–CNT–CNC—were fabricated and compared in terms of their transient electrical responses during NaCl-solution injection and subsequent natural drying. This sequential formulation comparison was designed to determine whether the overall electrical response changed following CNT incorporation and the subsequent addition of CNCs, rather than assigning independently verified functions to the individual additives. The morphologies and crystalline structures of the fabricated nanofiber films were examined using scanning electron microscopy and X-ray diffraction, respectively. Raman and FTIR spectroscopy were additionally used to examine CNT incorporation and changes in the chemical environment of the composite films. Three successive injection–drying cycles were conducted to observe repeated transient responses within individual devices. Because multiple independently fabricated devices and extended cycling tests were not evaluated, these measurements do not establish device-to-device reproducibility or long-term durability.

In our previous study, electrospun PAN–cellulose composite nanofibers were investigated for mechanically induced piezoelectric conversion [34]. The present study extends this material platform to a different operating condition by examining the transient electrical response produced during NaCl-solution absorption and subsequent drying without externally applied mechanical deformation. The emphasis is therefore placed on the temporal evolution and polarity of the wetting–drying-dependent electrical response rather than on optimized output power or quantitatively established energy-harvesting performance.

## 2. Materials and Methods

### 2.1. Materials

Polyacrylonitrile (PAN; average molecular weight, Mv = 150,000) was purchased from Sigma-Aldrich (St. Louis, MO, USA). N,N-Dimethylformamide (DMF, 99.8%) was supplied by Daejung Chemicals (Siheung, Republic of Korea) and used as the solvent for preparing the electrospinning solutions. An aqueous cellulose nanocrystal (CNC) slurry with a solid content of 10.4 wt% was supplied by the University of Maine (Orono, ME, USA).

Multi-walled carbon nanotubes (MWCNTs; CNT MR99) were obtained from Carbon Nano-material Technology Co., Ltd. (Republic of Korea). According to the manufacturer, the MWCNTs had diameters of 5–15 nm, with an average diameter of approximately 10 nm, lengths of approximately 10 μm, an aspect ratio greater than 500, and a purity greater than 99 wt%. Ultrapure water was purchased from Clover Chemical Co., Ltd. (Republic of Korea). Anhydrous ethyl alcohol (99.5%) and sodium chloride solutions with concentrations of 1, 2, and 5 wt% were purchased from Daejung Chemicals (Republic of Korea).

### 2.2. Preparation of CNC Powder

The as-received CNC material was initially supplied as an aqueous slurry containing 10.4 wt% CNC solids. To obtain solid CNC powder, the slurry was freeze-dried at −85 °C for 3 d using a freeze dryer (FD8508, ilShinBioBase Co., Ltd., Dongducheon-si, Republic of Korea). The resulting dried CNC solids were collected and weighed based on the required composition of the electrospinning solution.

### 2.3. Preparation of PAN-Based Electrospinning Solution

Three precursor solutions were prepared for the fabrication of pristine PAN, PAN–CNT, and PAN–CNT–CNC nanofiber films. The concentrations of PAN, MWCNTs, and CNCs were calculated relative to the total mass of each precursor solution. The PAN concentration was fixed at 10 wt% for all formulations. The PAN–CNT solution contained 0.1 wt% MWCNTs, whereas the PAN–CNT–CNC solution contained 0.1 wt% MWCNTs and 0.1 wt% freeze-dried CNC solids. The compositions of the three precursor solutions are summarized in Table 1.

For the pristine PAN solution, the required amount of PAN powder was added to DMF. The mixture was then heated to 50 °C and stirred at 500 rpm for 24 h until a homogeneous PAN solution was obtained.

For the PAN–CNT solution, the required amount of PAN powder was first added to DMF. While the mixture was stirred at 500 rpm and maintained at 50 °C to dissolve PAN, 0.1 wt% MWCNTs were introduced into the solution. Stirring was continued for a total of 24 h to complete PAN dissolution and promote the distribution of MWCNTs throughout the precursor solution.

For the PAN–CNT–CNC solution, PAN powder was first added to DMF. During the subsequent stirring and dissolution process at 50 °C and 500 rpm, 0.1 wt% MWCNTs and 0.1 wt% freeze-dried CNC solids were added to the mixture. The resulting precursor solution was stirred for a total of 24 h to complete PAN dissolution and promote the distribution of both nanofillers throughout the solution.

### 2.4. Fabrication of Electrospun Nanofiber Films

PAN-based nanofiber films were fabricated using an electrospinning procedure adapted from our previously reported method [34], with modifications to the precursor compositions for the preparation of PAN, PAN–CNT, and PAN–CNT–CNC films. Electrospinning was performed in an acrylic chamber maintained at 27 ± 1 °C; and 45 ± 2% relative humidity. Each precursor solution was loaded into a syringe equipped with a 22-gauge needle and delivered at a constant flow rate of 1 mL/h using a syringe pump.

The distance between the needle tip and the cylindrical drum collector was fixed at 15 cm. The collector, measuring 9 cm in diameter and 20 cm in length, was rotated at 300 rpm. An applied voltage of 18.5 kV was supplied using a high-voltage power source (SJ-5001P, Sejin Electronics Co., Seoul, Republic of Korea). For each composition, 2 mL of precursor solution was electrospun over 2 h.

After electrospinning, the films were vacuum-dried at 50 °C for 10 h to remove residual DMF before device fabrication and characterization.

### 2.5. Fabrication of the Sandwich-Type Electricity-Generating Device

A sandwich-type electricity-generating device was fabricated using two microscope slide-glass substrates, copper-tape electrodes, and an electrospun PAN-based nanofiber film. Two glass substrates were cut into rectangular pieces measuring 10 × 26 mm. Copper tape was attached to the inner surface of each substrate and extended beyond the edge of the glass to provide connection points for alligator clips.

The electrospun PAN, PAN–CNT, or PAN–CNT–CNC film was cut to 10 × 25 mm and placed between the two copper-tape-coated glass substrates. The substrates were aligned in a face-to-face configuration so that the copper electrodes contacted the opposite surfaces of the nanofiber film. The assembled structure was secured using clips to maintain consistent contact between the electrodes and the film. For electrical measurements, separate alligator clips were connected to the extended portions of the upper and lower copper-tape electrodes.

### 2.6. Characterization

The crystalline structures of the electrospun films were characterized using an X-ray diffractometer (X’Pert PRO MPD, Malvern Panalytical) equipped with a Cu Kα radiation source at the Korea Basic Science Institute, Western Seoul Center, Republic of Korea. The surface morphologies of the films were examined using a field-emission scanning electron microscope (CLARA LMH, TESCAN, Brno, Czech Republic) after sputter coating with Au. Raman spectra of the electrospun films were acquired using an AFM–Raman system (NTEGRA Spectra, NT-MDT Spectrum Instruments, Moscow, Russia). Fourier-transform infrared (FTIR) spectra were obtained using an attenuated-total-reflectance FTIR spectrometer (IdentifyIR, Smiths Detection, Watford, UK).

The open-circuit voltage and short-circuit current responses were recorded using a digital multimeter (DMM6500, Keithley Instruments, Solon, OH, USA). For each measurement, 20 μL of the designated solution was deposited at the same predefined position on the exposed portion of the nanofiber film and allowed to spread through the porous membrane. During repeated injection–drying measurements, the device was allowed to dry naturally, and the next injection was performed after the preceding voltage response had approached its baseline. For the formulation comparison in Figure 2, one device per composition was subjected to three successive injection–drying cycles. For the preliminary interconnection tests, 20 μL of solution was separately supplied to each constituent device.

## 3. Results and Discussion

**Figure 1:**
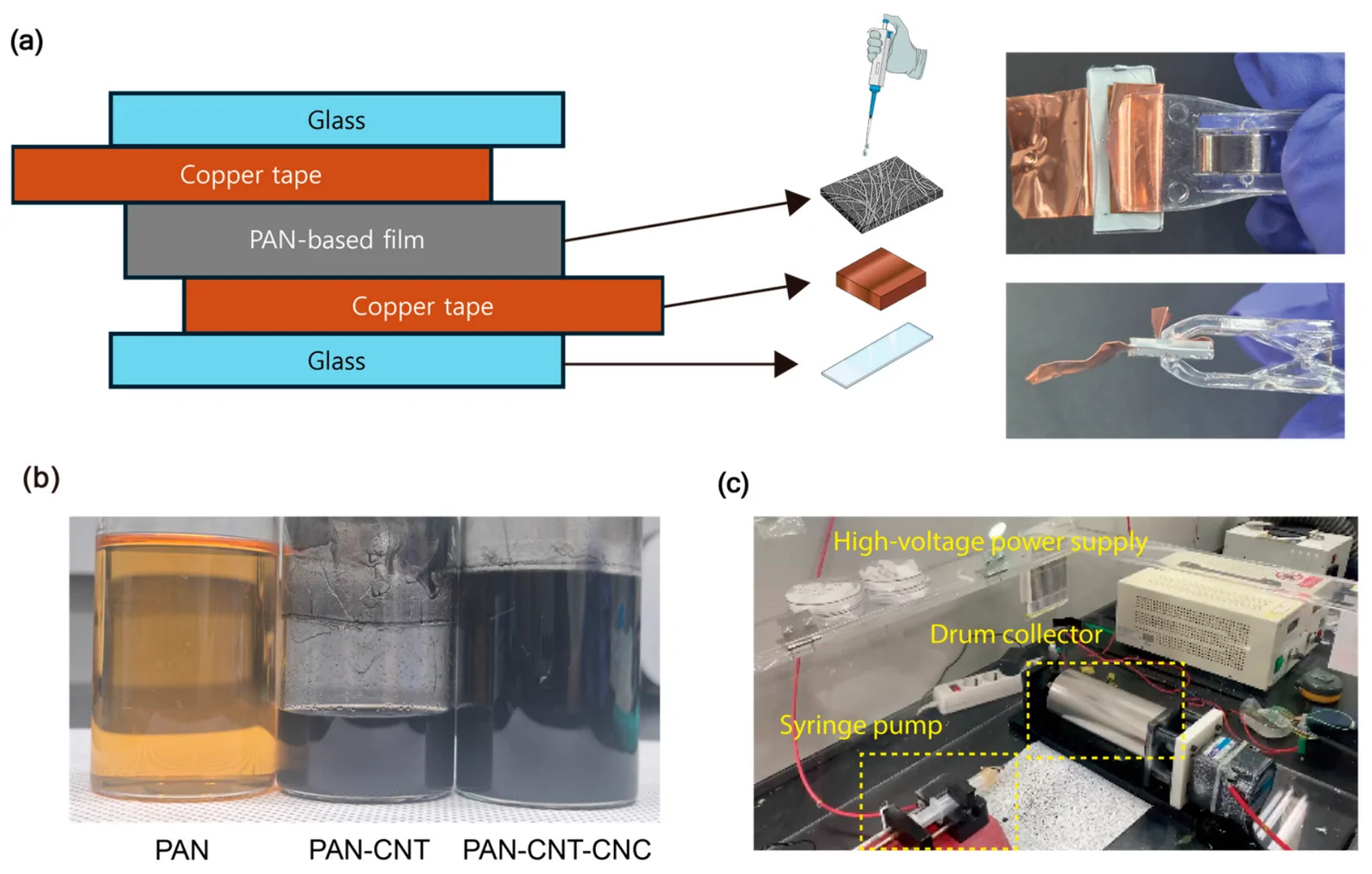
Fabrication process and operating principle of the PAN-based nanofiber electricity generator: (**a**) schematic illustration of the device structure and photograph of the fabricated device, (**b**) precursor solutions prepared for electrospinning (PAN, PAN–CNT, and PAN–CNT–CNC), and (**c**) photograph of the electrospinning setup.

Figure 1 illustrates the fabrication process and operating principle of the PAN-based nanofiber electricity generator. Three precursor compositions, namely pristine PAN, PAN–CNT, and PAN–CNT–CNC, were prepared and electrospun under identical processing conditions to form porous nanofiber films. The electrospun films were subsequently vacuum-dried and cut into rectangular specimens measuring 10 × 25 mm.

Each nanofiber film was placed between two copper-tape-coated glass substrates measuring 10 × 26 mm to form a sandwich-type electricity-generating device. The upper and lower copper electrodes contacted the opposite surfaces of the film, and the assembled device was secured using clips. The copper tapes were extended beyond the glass substrates to enable electrical connection using alligator clips.

A transient electrical response was initiated by depositing a small quantity of NaCl solution on the exposed portion of the porous nanofiber film. The solution was absorbed into and spread through the porous membrane, producing a measurable response between the two electrodes. The electrical responses of the PAN, PAN–CNT, and PAN–CNT–CNC devices were subsequently compared using a 2 wt% NaCl solution under the same nominal injection conditions.

**Figure 2:**
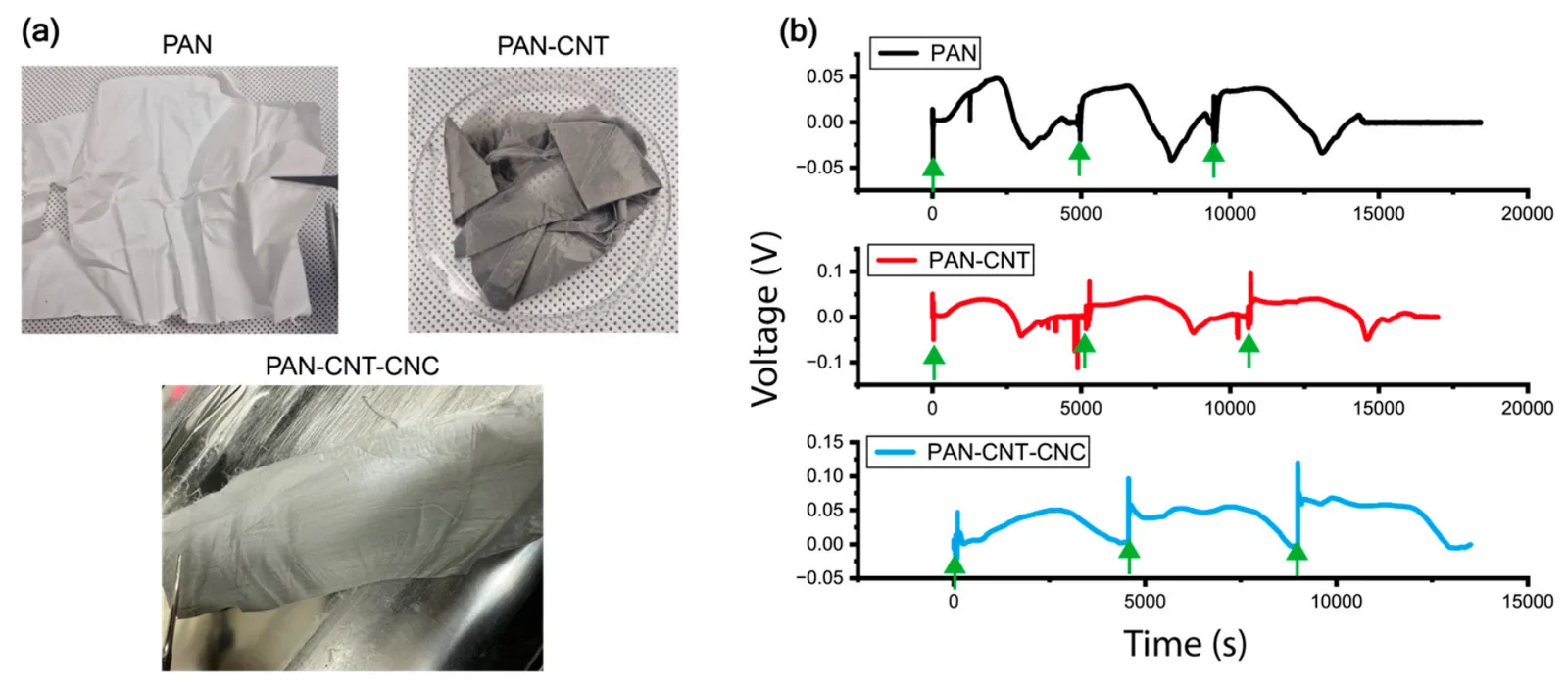
(**a**) Photographs of the electrospun PAN, PAN–CNT, and PAN–CNT–CNC nanofiber films. (**b**) Voltage responses of the corresponding devices during three successive injection–drying cycles using a 2 wt% NaCl solution. The green arrows indicate the NaCl-solution injection times.

Figure 2a shows the large-area PAN, PAN–CNT, and PAN–CNT–CNC nanofiber films fabricated by electrospinning. Figure 2b compares the time-dependent voltage responses of the corresponding electricity-generating devices during three successive injection–drying cycles using a 2 wt% NaCl aqueous solution. The controlled NaCl solution was employed as a model saline electrolyte to compare the effect of nanofiber-film composition under identical electrolyte conditions [35].

For each device, the NaCl solution was introduced three times. After each injection, the solution was absorbed into the porous nanofiber film and a measurable voltage response was observed. The devices were then allowed to dry naturally without additional solution supply. As water gradually evaporated, the voltage approached its pre-injection baseline, after which the next injection was performed. Because residual NaCl remained in the porous membrane after drying, the successive cycles did not begin from the original salt-free state. The protocol was therefore used to observe repeated transient responses within an individual device rather than complete regeneration or long-term cycling stability.

As shown in Figure 2, all three devices exhibited renewed voltage generation after each NaCl-solution injection, indicating that the electrical output was associated with solution infiltration into the porous nanofiber films. The maximum open-circuit voltages of the PAN, PAN–CNT, and PAN–CNT–CNC devices were approximately 47, 42, and 68 mV, respectively. These values represent the maximum post-injection voltage responses, excluding the brief spikes occurring at the injection events. The spikes remain visible in Figure 2b. Among the individual devices tested, the PAN–CNT–CNC device exhibited the highest peak voltage. The voltage-decay and negative-voltage behaviors also differed considerably among the three devices.

The pristine PAN device exhibited a progressive decrease in the positive-voltage response over the three successive injection–drying cycles. A relatively pronounced and prolonged negative-voltage region was also observed during the voltage-decay stage. This polarity reversal may reflect relaxation of the nonequilibrium ionic and interfacial charge distributions established during solution absorption. Ion back-diffusion, capacitive discharge, electrode polarization, nonuniform drying, and baseline drift may also contribute to the observed reverse transient. Because the present measurements do not directly resolve the spatial and temporal distributions of ionic or electronic charges, the negative-voltage response is interpreted as an experimentally observed transient phenomenon rather than definitive evidence of a specific charge-transfer mechanism.

The PAN–CNT device also exhibited repeated transient voltage responses over the three injection–drying cycles, while the magnitude and duration of its negative-voltage region were smaller than those observed for the pristine PAN device. CNTs have been reported to possess favorable charge-transfer characteristics and to serve as solid-state electron mediators in other interfacial energy-conversion systems [28]. However, the operating mechanism of the cited system differs from the present NaCl-solution infiltration process. Moreover, the PAN–CNT device did not exhibit a higher maximum voltage than the pristine PAN device, and the electrical conductivity, sheet resistance, and impedance of the membranes were not measured. Therefore, Reference [28] provides a material-design rationale for CNT incorporation but does not demonstrate that 0.1 wt% CNTs formed a continuous conductive network or improved charge transport in the present device.

Among the three tested formulations, the PAN–CNT–CNC device exhibited the largest maximum voltage in the corresponding experiment and showed repeated transient responses during the three successive injection–drying cycles. The magnitude of the negative-voltage response was also smaller than that observed for the pristine PAN device. Previous studies have reported that cellulose–carbon nanomaterial composites can exhibit composition- and structure-dependent electrical and interfacial characteristics [31,32]. These studies provide a general material context for examining the PAN–CNT–CNC formulation but do not establish that the present response resulted from complementary or synergistic functions of CNTs and CNCs. Because a PAN–CNC control, quantitative wetting measurements, and membrane-conductivity measurements were not available, the individual contribution of CNCs and the respective effects of liquid absorption, membrane morphology, interfacial interactions, and electrical transport cannot be separated from the present data.

A comparison with a Cu/filter-paper/Cu control prepared and measured under comparable conditions is provided in Figure S2. Under the tested conditions, the filter-paper control did not reproduce the characteristic transient response observed in the PAN device. This comparison does not independently exclude contributions from electrode polarization or Cu/electrolyte interactions.

Overall, the three successive injection–drying cycles demonstrate that transient voltage responses were repeatedly observed after NaCl-solution injection into the tested devices. However, residual NaCl remained within the porous membranes after drying; therefore, the subsequent cycles did not begin from the original salt-free state and should not be interpreted as fully regenerated operating cycles. The PAN–CNT–CNC formulation was selected for subsequent characterization and preliminary device experiments because it exhibited the largest maximum voltage and a clearly observable repeated transient response under the tested conditions. These observations apply to the individual devices examined in this study and do not establish device-to-device reproducibility, long-term cycling stability, or statistically significant superiority among the formulations.

**Figure 3:**
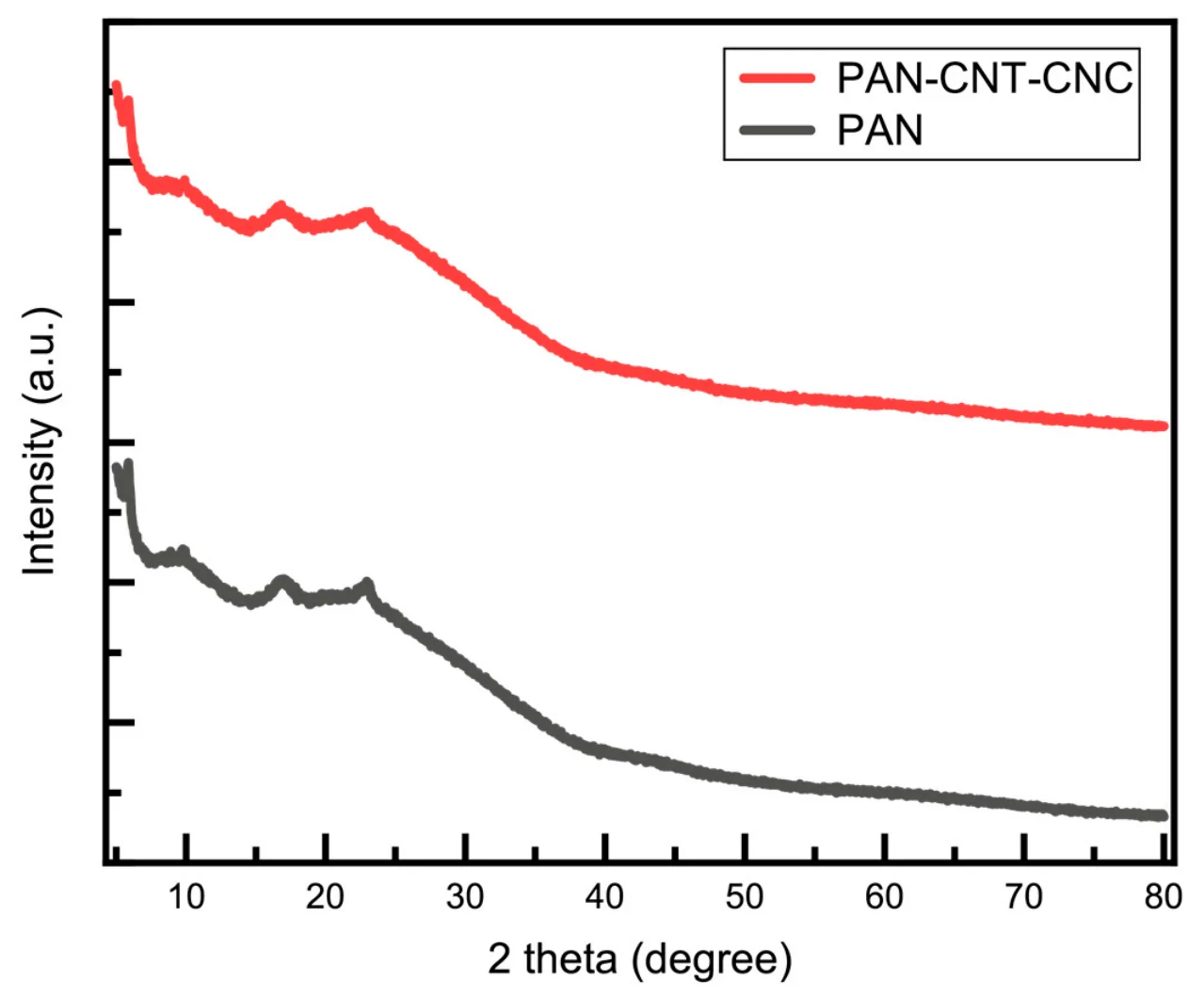
X-ray diffraction patterns of the PAN and PAN–CNT–CNC nanofiber films.

Figure 3 compares the X-ray diffraction (XRD) patterns of the pristine PAN and PAN–CNT–CNC nanofiber films. Both samples exhibited the characteristic broad diffraction features of PAN-based polymer films, indicating that the dominant structural characteristics of the PAN matrix were retained after the incorporation of CNTs and CNCs. No prominent additional diffraction peaks attributable to secondary crystalline phases were observed in the PAN–CNT–CNC film.

Distinct diffraction peaks corresponding to CNTs and CNCs were not clearly resolved, which may be attributed to their low concentrations of 0.1 wt% each, overlap with the broad PAN diffraction features, and the limited detectability of highly dispersed nanofillers by conventional XRD. Overall, the XRD results indicate that the incorporation of CNTs and CNCs did not substantially alter the PAN-based structure or produce detectable crystalline impurity phases.

**Figure 4:**
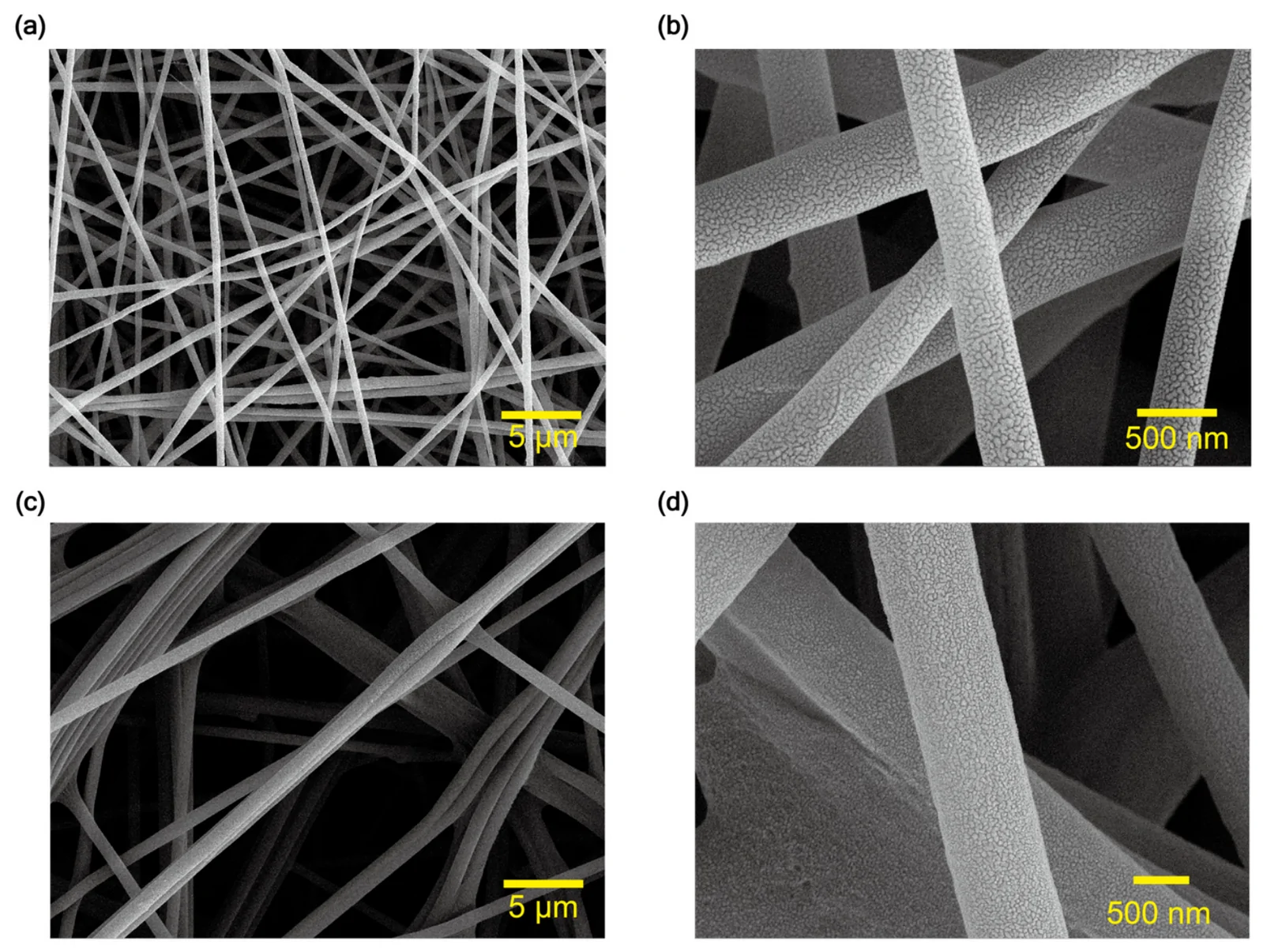
SEM images of the electrospun (**a**,**b**) PAN and (**c**,**d**) PAN–CNT–CNC nanofiber films at different magnifications.

Figure 4 presents scanning electron microscopy (SEM) images of the electrospun pristine PAN and PAN–CNT–CNC nanofiber films at different magnifications. Both films consisted of continuous and interconnected nanofibers with open void spaces throughout the networks, confirming the successful formation of porous nanofiber structures by electrospinning. The nanofibers were distributed relatively uniformly over the observed regions and exhibited smooth surfaces without prominent bead formation.

The interconnected porous morphology provides a high surface-to-volume ratio and accessible pathways for NaCl-solution absorption, allowing contact between the electrolyte and the nanofiber surfaces. Because the SEM analysis was performed on dry films, however, the images confirm only the qualitative presence of porous nanofiber networks and do not establish equivalent fiber-diameter distributions, membrane thicknesses, porosities, liquid-transport behavior, or interfacial charge-transfer properties among the formulations.

Overall, the SEM images qualitatively show that both the pristine PAN and PAN–CNT–CNC samples retained continuous, interconnected fibrous morphologies under the applied electrospinning conditions. Possible differences in membrane architecture and their contributions to liquid absorption and electrical response cannot be excluded without quantitative morphological and structural measurements.

**Figure 5:**
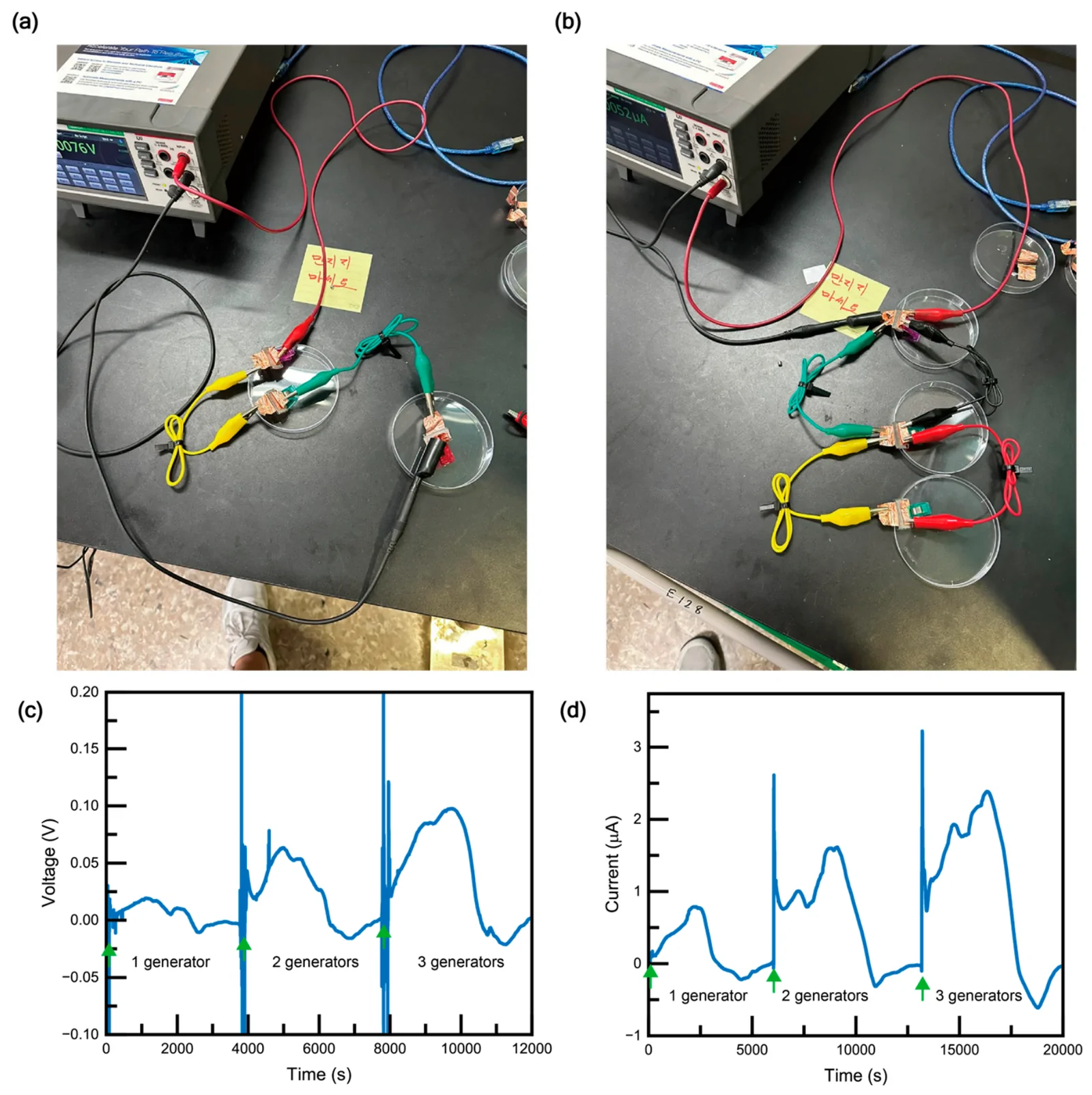
Preliminary interconnection tests of PAN–CNT–CNC devices: (**a**) three devices connected in series, (**b**) three devices connected in parallel, (**c**) voltage responses for one to three series-connected devices, and (**d**) current responses for one to three parallel-connected devices. The green arrows indicate the NaCl-solution injection times.

Preliminary interconnection tests were performed by sequentially increasing the number of connected PAN–CNT–CNC devices from one to three. Figure 5a and Figure 5b show the series and parallel configurations, respectively. Figure 5c presents the voltage measured during the series-connection sequence, whereas Figure 5d presents the current measured during the parallel-connection sequence. Series-connected current and parallel-connected voltage were not measured in this preliminary experiment.

In the single-device stage, Device 1 underwent its first solution injection. In the two-device stage, Device 1 had already experienced one injection–drying step, whereas Device 2 underwent its first injection. In the three-device stage, Devices 1, 2, and 3 had experienced two, one, and zero previous injection–drying steps, respectively. Consequently, the constituent devices did not have identical initial wetting states, residual-ion distributions, or drying histories during the sequential test.

The measured series voltage changed from approximately 19 mV for one device to 63 mV for two devices and 97 mV for three devices, while the measured parallel current changed from approximately 0.786 μA to 1.592 μA and 2.383 μA, respectively. Because the individual output of each constituent device was not measured under the same initial condition immediately before interconnection, these values cannot be compared quantitatively with ideal mathematical scaling. Non-ideal stack behavior has also been examined in electrochemical systems [36]; however, the operating principle of the cited electrolyzer system differs from that of the present device and does not explain the deviations observed here. The present results are therefore reported only as a preliminary qualitative demonstration that the measured voltage or current changed as additional devices were connected.

**Figure 6:**
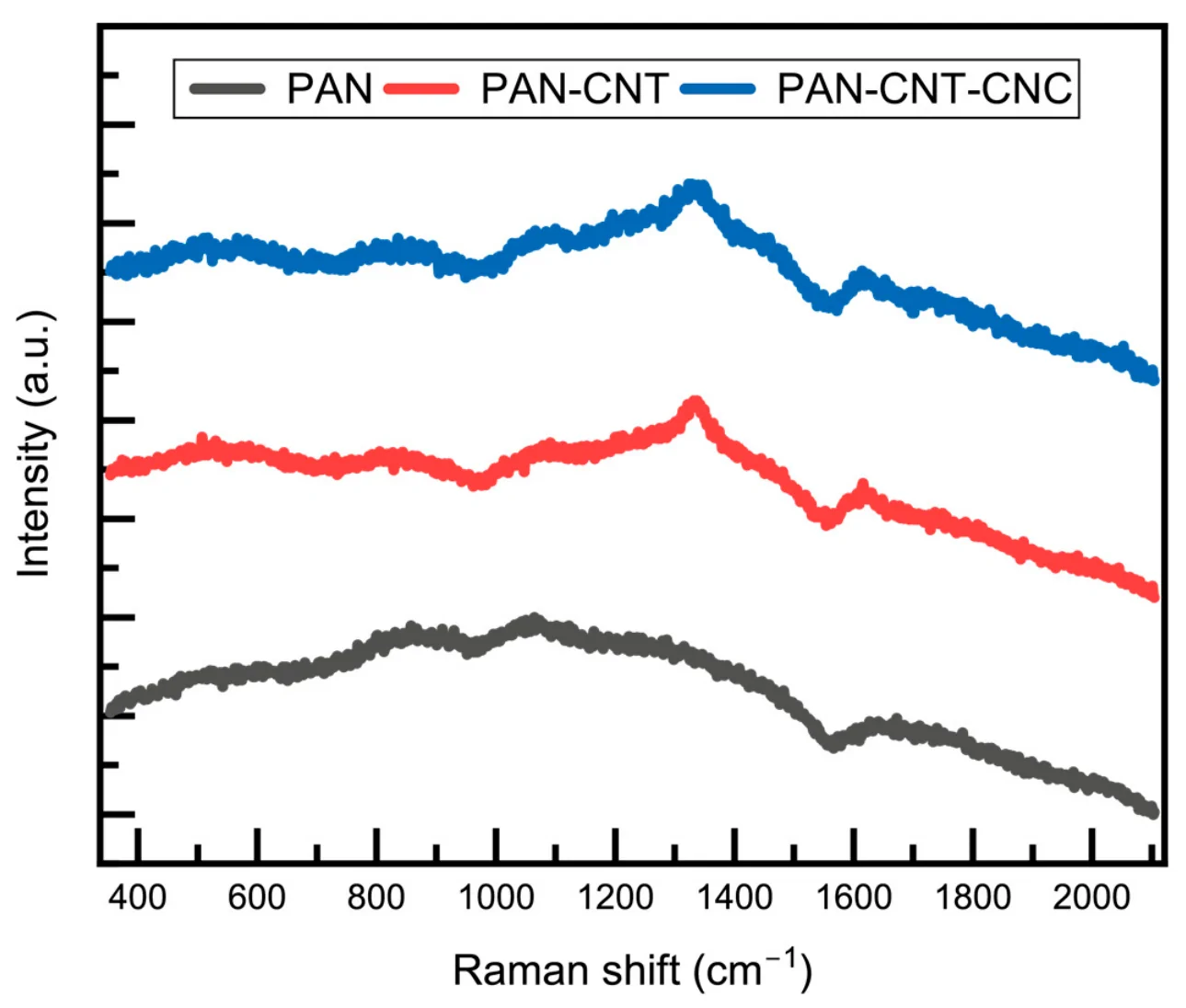
Raman spectra of the electrospun pristine PAN, PAN–CNT, and PAN–CNT–CNC nanofiber films. CNT-associated spectral features in the D- and G-band regions are observed in the PAN–CNT and PAN–CNT–CNC samples. The spectra are vertically offset for clarity.

The Raman spectra of the PAN, PAN–CNT, and PAN–CNT–CNC nanofiber films are presented in Figure 6. Compared with the pristine PAN film, the PAN–CNT and PAN–CNT–CNC films exhibited additional spectral features in the characteristic D- and G-band regions of CNTs. The D band is associated with defect- and disorder-induced scattering in graphitic carbon, whereas the G band originates from the in-plane vibration of sp^2^-bonded carbon atoms. The appearance of these characteristic bands in both CNT-containing samples provides spectroscopic evidence for the successful incorporation of CNTs into the electrospun PAN-based nanofiber films [37].

The Raman measurements were used primarily to confirm the presence of CNTs. Because spatially resolved Raman mapping was not performed, these spectra do not provide a quantitative evaluation of CNT dispersion throughout the entire nanofiber film. Therefore, no conclusion regarding the quantitative dispersion uniformity of CNTs was drawn from the Raman results.

**Figure 7:**
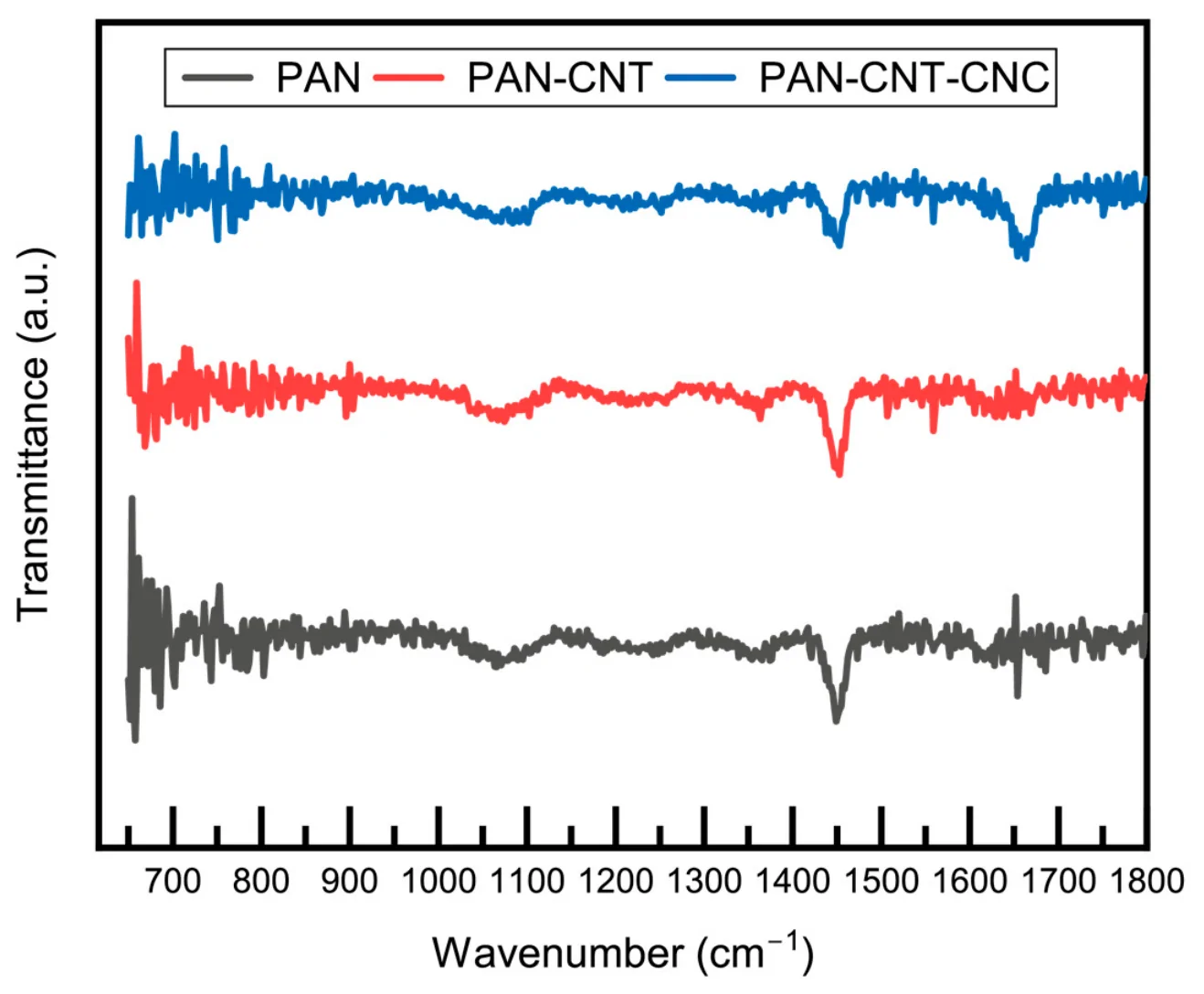
FTIR transmittance spectra of the electrospun pristine PAN, PAN–CNT, and PAN–CNT–CNC nanofiber films. The characteristic PAN absorption region near 1450 cm^−1^ and the absorption feature near 1646 cm^−1^ observed in the PAN–CNT–CNC film are indicated. The spectra are vertically offset for clarity.

Figure 7 presents the FTIR transmittance spectra of the PAN, PAN–CNT, and PAN–CNT–CNC nanofiber films. All three samples retained the principal characteristic absorption bands of the PAN matrix, indicating that the fundamental chemical structure of PAN was preserved after the incorporation of CNTs and CNCs. The absorption band near 1450 cm^−1^, observed in all three samples, was primarily assigned to the bending vibration of the –CH_2_– groups in the PAN backbone [38].

In contrast, a distinct absorption feature near 1646 cm^−1^ was observed in the PAN–CNT–CNC film and was not clearly detected in either the pristine PAN or PAN–CNT film. This region is commonly associated with the H–O–H bending vibration of adsorbed or bound water and may be influenced by the hydroxyl-rich CNC component [39]. Its selective appearance suggests a change in the water-associated chemical environment of the PAN–CNT–CNC membrane. This observation is consistent with the qualitative difference in water spreading and absorption shown in Supplementary Videos S1 and S2, but it does not quantitatively establish CNC content, enhanced hydrophilicity, or uniform liquid infiltration.

The feature near 1646 cm^−1^ is therefore interpreted cautiously as evidence of a changed water-interacting environment rather than the formation of a new covalent bond between CNCs and PAN. Taken together, Raman and FTIR provide complementary analytical observations supporting CNT incorporation and a change in the water-associated environment of the PAN–CNT–CNC membrane, respectively; they do not establish CNT dispersion uniformity, CNC content, or synergistic interactions between the additives.

Accordingly, Raman spectroscopy supported the incorporation of CNTs through their characteristic graphitic bands, whereas FTIR indicated a change in the water-associated chemical environment of the PAN–CNT–CNC membrane. These spectroscopic observations suggest a modified water-associated environment in the PAN–CNT–CNC membrane but do not establish that this modification caused the observed electrical response. The absence of a PAN–CNC control is a limitation of the present study, leaving the contribution of CNCs in the absence of CNTs and any CNT–CNC interaction unresolved.

Overall, the preliminary interconnection experiment showed changes in the measured voltage and current as the number of connected PAN–CNT–CNC devices was increased. However, the sequential protocol involved devices with different injection–drying histories, and the output of each constituent device was not independently measured under identical initial conditions. The results therefore do not establish ideal series or parallel scaling, quantitative connection efficiency, or practical modular performance. Load-dependent power output, internal resistance, long-term operation, and device-to-device reproducibility remain to be evaluated.

**Figure 8:**
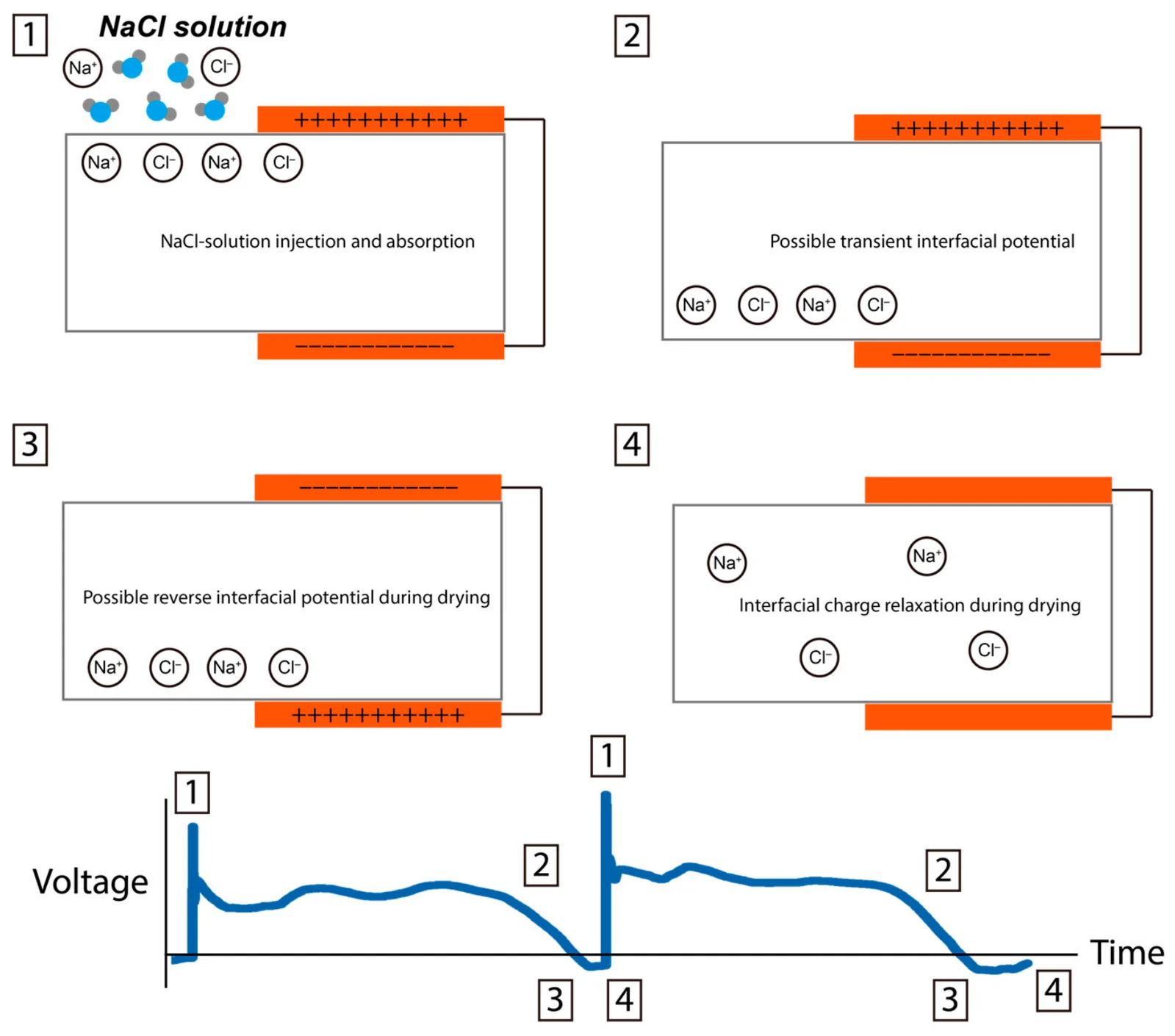
Schematic illustration of the proposed phenomenological interpretation of the transient electrical response during NaCl-solution absorption and drying in the PAN–CNT–CNC nanofiber film.

Figure 8 presents a phenomenological interpretation of the transient electrical response observed during NaCl-solution absorption and subsequent drying in the porous PAN–CNT–CNC nanofiber film. The discussion is informed by previously reported water-infiltration-induced ion–surface interactions in porous structures [18], but the microscopic mechanism of the present polymer-composite device was not directly measured.

Following deposition, the NaCl solution is absorbed into and spreads through the porous nanofiber network. During this time-dependent transition, dissolved Na^+^ and Cl^−^ ions interact with the membrane and electrode interfaces. Changes in local water content, ion concentration, differential ion adsorption, and electrical-double-layer formation may produce a transient nonequilibrium charge distribution and a measurable potential difference between the electrodes.

During subsequent drying, evaporation changes the local ion concentration, mobility, and interfacial conditions. Redistribution of mobile ions and relaxation of accumulated interfacial charge may contribute to the reverse-voltage transient. Electrode polarization, capacitive discharge, nonuniform drying, and baseline drift may also contribute. Previous work on porous CuO nanowire films reported water-infiltration-induced ionovoltaic generation using 20 μL of 0.1 M NaCl solution [18]; however, the inorganic semiconductor system and its verified mechanism differ from the present PAN-based composite membrane.

The inclusion of CNTs and CNCs changes the overall formulation and may also affect membrane morphology, liquid absorption, interfacial properties, and electrical transport. However, membrane conductivity, quantitative wetting behavior, surface potential, ion adsorption, carrier concentration, and spatial ion distributions were not measured. The individual roles of CNTs and CNCs and any possible contribution from CNT-mediated electronic transport therefore cannot be separated from the present observations.

Accordingly, the observed response is discussed phenomenologically in relation to time-dependent liquid absorption, ion redistribution, interfacial charge relaxation, electrode polarization, and capacitive effects. The relative contribution of each process remains unresolved, and Figure 8 should not be interpreted as a directly verified microscopic charge-transport model.

**Figure 9:**
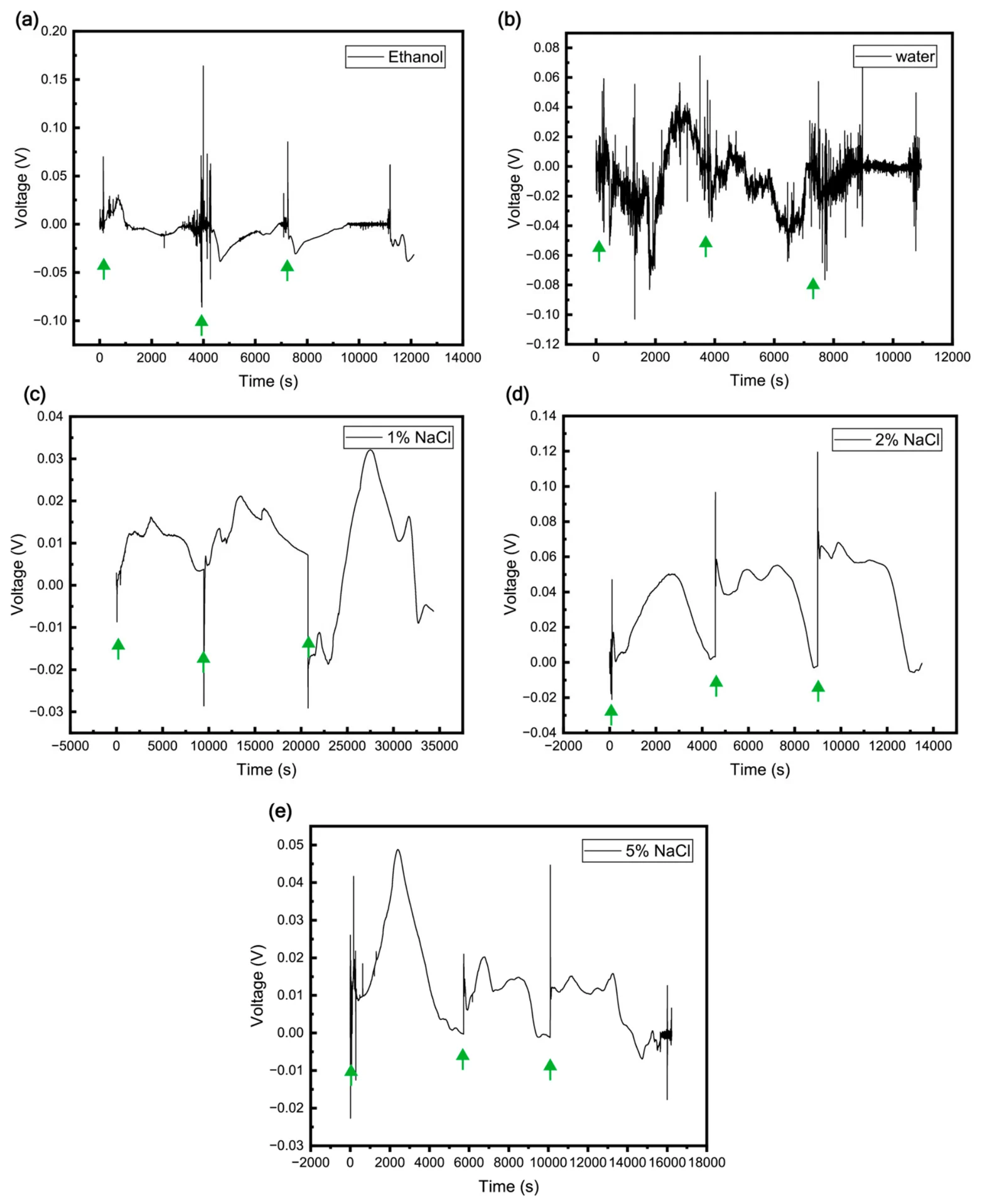
Voltage responses of the PAN–CNT–CNC device during three successive injection–drying cycles using (**a**) ethanol, (**b**) deionized water, (**c**) 1 wt% NaCl solution, (**d**) 2 wt% NaCl solution, and (**e**) 5 wt% NaCl solution. The voltage response in each panel is presented relative to the corresponding pre-injection baseline, and the injection times are indicated by green arrows.

Figure 9 compares the time-dependent voltage responses of the PAN–CNT–CNC device during three successive injection–drying cycles using ethanol, deionized water, and 1, 2, and 5 wt% NaCl solutions, as shown in Figure 9a–e, respectively. For each cycle, 20 μL of the designated liquid was introduced after the preceding response had approached its baseline through natural drying. The green arrows indicate the liquid-injection times. Because residual solute could remain after evaporation, the successive cycles did not represent identical initial conditions. The comparison among the liquids is therefore based on the injection-induced voltage change relative to the corresponding pre-injection baseline.

The 1 wt% NaCl solution produced a smaller injection-induced voltage change than the 2 wt% solution in the tested device. Na^+^ and Cl^−^ ions may participate in interfacial ion adsorption, electrical-double-layer formation, and time-dependent charge redistribution within the wetted porous membrane. At the lower concentration, the availability of mobile ions and the resulting interfacial response may differ from those under the 2 wt% condition. However, the present measurements do not directly resolve the electrical double layer or the spatial ion distribution.

Among the tested conditions, the 2 wt% NaCl solution produced the largest injection-induced voltage change in the corresponding experiment. This observation may reflect concentration-dependent differences in mobile-ion availability, interfacial ion adsorption, ionic transport, and electrostatic screening within the porous PAN–CNT–CNC nanofiber membrane. Because only an individual device and three successive cycles were examined, this result does not establish statistical superiority, device-to-device reproducibility, or an optimized electrolyte concentration.

Increasing the NaCl concentration to 5 wt% produced a smaller injection-induced voltage change than the 2 wt% condition in the tested device. This non-monotonic response indicates that the measured voltage was not determined solely by bulk ionic conductivity or the total amount of dissolved salt. At elevated concentrations, electrostatic screening, ion–ion interactions, and changes in interfacial double-layer structure may affect the transient potential response, as reported for other electrolyte and porous-electrode interfaces [40,41,42]. However, these processes were not directly measured in the present device.

The injection-induced voltage changes obtained with deionized water and ethanol were smaller than those observed with the aqueous NaCl solutions. These liquids differ in mobile-ion content, dielectric properties, wetting and absorption behavior, and evaporation characteristics. Such differences may influence the transient interfacial response, but the present data do not permit the individual contribution of each property to be determined.

Overall, the dependence of the transient voltage response on the liquid type and nominal NaCl concentration is consistent with a contribution from dissolved ions and time-dependent interfacial processes. Nevertheless, electrical-double-layer formation, selective ion adsorption, electrostatic screening, and ion redistribution were not directly measured. The concentration-dependent observations are therefore presented as phenomenological trends in the tested device rather than as definitive verification of a specific microscopic mechanism.

## 4. Conclusions

This study examined the transient electrical response of electrospun PAN-based porous composite membranes during NaCl-solution injection and subsequent drying. Different voltage waveforms were observed among the tested PAN, PAN–CNT, and PAN–CNT–CNC devices, with reverse-voltage transients observed during drying. Raman analysis supported CNT incorporation, while FTIR and supplementary droplet-absorption observations indicated changes in the water-interacting behavior of the PAN–CNT–CNC membrane. However, the individual contribution of CNCs, CNT-network conductivity, and the microscopic origin of the electrical response were not independently established.

The three successive injection–drying cycles demonstrate repeated transient responses within the tested devices but do not establish device-to-device reproducibility or long-term durability. Likewise, the preliminary interconnection tests do not provide a quantitative evaluation of ideal series or parallel scaling. The principal contribution of this work is therefore the observation of wetting–drying-dependent electrical behavior in an electrospun PAN-based membrane platform. Load-dependent power measurements, quantitative morphology and wetting analyses, inert-electrode controls, complete compositional controls, and long-term cycling tests are required to evaluate the mechanism and practical energy-harvesting capability.

## Figures and Tables

**Table 1:** Composition of the PAN-based electrospinning solutions.

Sample	PAN Content	CNT Content	CNC Content
PAN	10 wt%	–	–
PAN–CNT	10 wt%	0.1 wt%	–
PAN–CNT–CNC	10 wt%	0.1 wt%	0.1 wt%

## Data Availability

The data presented in this study are contained within the article and its Supplementary Materials and Video S1, S2.

## Supplementary Materials

The following supporting information can be downloaded at: https://www.mdpi.com/article/10.3390/polym18192430/s1, Figure S1: SEM images of the electrospun PAN–CNT nanofiber films at different magnifications; Figure S2: Comparison of the voltage responses of Cu/PAN/Cu and Cu/filter-paper/Cu devices under the same NaCl-solution injection protocol; Figure S3: EDS analysis of the pristine PAN nanofiber film; Figure S4: EDS analysis of the PAN–CNT–CNC nanofiber film; Video S1: Water-droplet spreading and absorption on the pristine PAN nanofiber film; Video S2: Water-droplet spreading and absorption on the PAN–CNT–CNC nanofiber film.

## Abbreviations

The following abbreviations are used in this manuscript:

PAN	polyacrylonitrile
CNT	carbon nanotube
CNC	cellulose nanocrystal
MWCNT	multi-walled carbon nanotube
DMF	N,N-dimethylformamide
XRD	X-ray diffraction
SEM	scanning electron microscopy
EDL	electric double layer
