# Supplementary material for: Fabrication of Electrospun PAN–CNT–CNC Composite Nanofiber Films for Hydrovoltaic Energy Harvesting
Source: Polymers (Basel). 2026 Oct 6;18(19):2430. doi: 10.3390/polym18192430 (PMC13667168; doi:10.3390/polym18192430)
Supplement: Supplementary file 1 [file polymers-18-02430-s001.zip › polymers-4487285-supplementary.pdf]

Supplementary Materials

Fabrication of Electrospun PAN–CNT–CNC Composite Nanofiber Films for Hydrovoltaic Energy Harvesting

Hee Chang Jeon, Woohyuk Jang, Jiseon Yun, Sein Min, Joong Yeon Lim * and Young-Seong Kim *

Department of Mechanical, Robotics and Energy Engineering, Dongguk University, Jung-gu, Seoul 04620, Republic of Korea; hcjeon@dongguk.edu (H.C.J.); jangwh21270@dgu.ac.kr (W.J.); ilyoung0430@dgu.ac.kr (J.Y.); molang0721@dgu.ac.kr (S.M.)

* Correspondence: jylim@dongguk.edu (J.Y.L.); kys4865@dgu.ac.kr (Y.-S.K.)

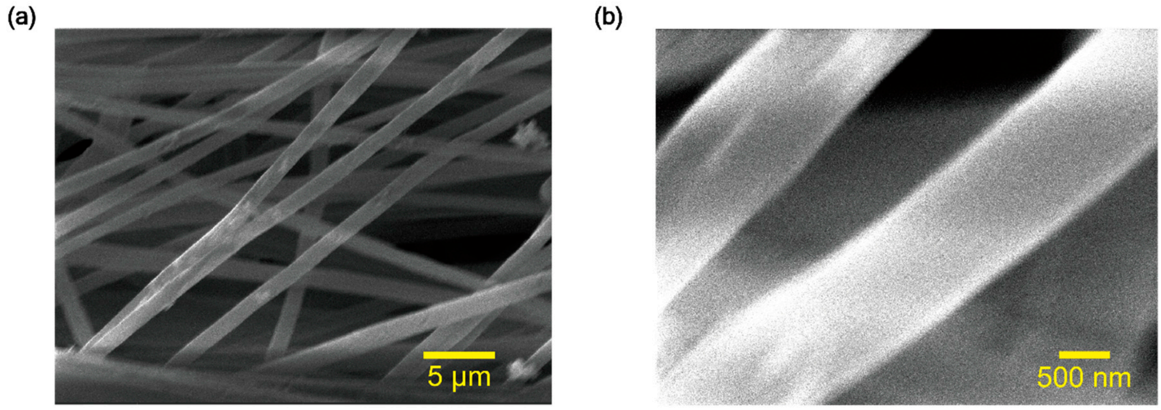

Figure S1. SEM images of the electrospun PAN-CNT nanofiber films at different magnifications

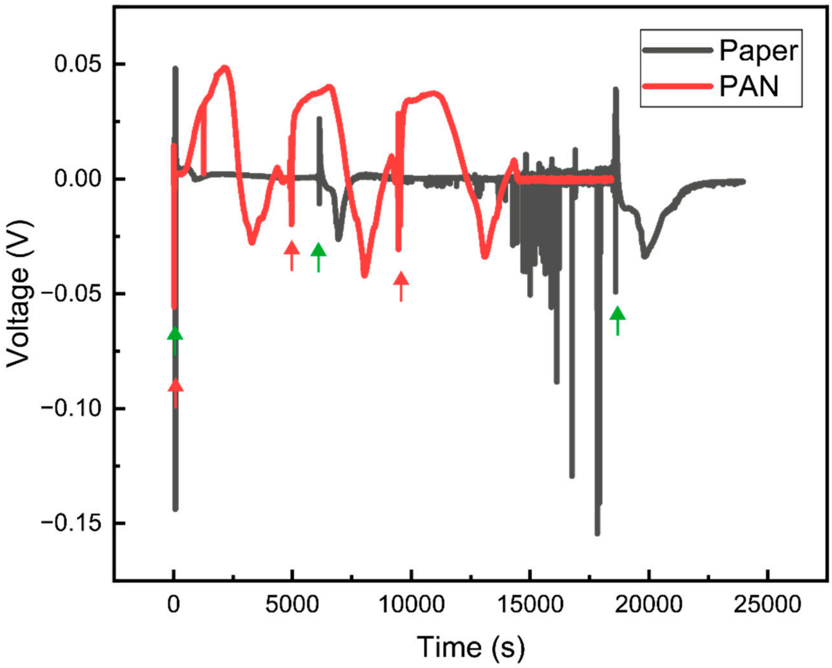

Figure S2. Comparison of the voltage responses of Cu/PAN/Cu and Cu/filter-paper/Cu devices under the same NaCl-solution injection protocol. The traces are overlaid using common axes and the same baseline treatment. Red and green arrows indicate injection events for the PAN and filter-paper devices, respectively.

Spectrum 1

Element	At. No.	Mass [%]	Mass Norm. [%]	Atom [%]	abs. error [mass%] (3 σ)
C	6	---	64.49	67.93	8.97
N	7	---	35.51	32.07	7.00
		**Sum**	**100.00**	**100.00**	

Figure S3. EDS analysis of the pristine PAN nanofiber film.

Spectrum 1

Element	At. No.	Mass [%]	Mass Norm. [%]	Atom [%]	abs. error [mass%] (3 σ)
C	6	---	66.98	70.29	9.33
N	7	---	33.02	29.71	6.84
		**Sum**	**100.00**	**100.00**	

Figure S4. EDS analysis of the PAN–CNT–CNC nanofiber film.
